# Advances in surgical techniques for fetal repair of myelomeningocele

**DOI:** 10.3171/2026.1.FOCVID2563

**Published:** 2026-04-01

**Authors:** Nalin Gupta, Robin Bowman, Abhaya V. Kulkarni, William Whitehead, Marike Zwienenberg

**Affiliations:** 1 Department of Neurological Surgery, University of California, San Francisco, California;; 2 Department of Neurosurgery, Northwestern University, Chicago, Illinois;; 3 Division of Neurosurgery, Hospital for Sick Children, University of Toronto, Ontario, Canada;; 4 Department of Neurosurgery, Baylor College of Medicine, Houston, Texas; and; 5 Department of Neurological Surgery, University of California Davis, Sacramento, California

## Introduction

In general, technical advances in surgical disciplines have followed a slow and incremental path toward more efficient and effective approaches. Less commonly, an advance has led to rapid changes in conventional treatment of a well-known condition. Myelomeningocele, the most common type of open neural tube defect, has been treated by neurosurgeons in a consistent manner for most of the 20th century. The basic principles, isolation of the neural placode and a layered closure of soft tissues, did not change for many years.

During the 1990s, an improved understanding of maternal physiology during pregnancy and the development of safe surgical approaches to the fetus led to a rapid expansion of prenatal conditions that could be treated in utero. Most of these conditions (e.g., sacrococcygeal teratoma) could lead to fetal demise, and therefore the risks of surgical intervention were less than those of the natural history of the disease. Children with myelomeningoceles, however, were usually delivered at term and, despite defined neurological deficits, could have long lifespans. The question was whether repair of the physical defect earlier in gestation would improve long-term outcome, and if that theoretical improvement was balanced by the associated risks.

Unlike many new surgical techniques, fetal repair of myelomeningocele was compared to a standard postnatal repair by a randomized clinical trial (Management of Myelomeningocele Study [MOMS]) that had a number of important features intended to reduce the impact of bias, particularly how it could impact the measurement of endpoints such as hydrocephalus and neurological function.^1^ Conducted at three centers in the United States, the group randomized to fetal surgery had reduced rates of hydrocephalus, reduction in the imaging appearance of Chiari type II malformation, and modest improvement in neurological function. Maternal and fetal risks, which were not inconsequential, were also clearly identified.

The results of the MOMS trial led to widespread adoption of the open hysterotomy technique, but also prompted rapid evolution and innovation of new technical approaches. The most important was the use of endoscopic tools to minimize material risks such as uterine rupture and premature delivery.^2^ The videos in this volume of *Neurosurgical Focus: Video* highlight the diversity of surgical techniques being used by many groups in the current era.

Myelomeningoceles demonstrate anatomical variations that can affect the ability to close various tissue planes primarily. Reilly et al. and Amaral-Nieves et al. demonstrate approaches that utilize mobilization of adjacent tissues to allow closure of the skin defect. Hersh et al. focus on the highly important step of uterine closure in order to prevent bleeding and premature rupture of membranes, which can greatly increase maternal morbidity. Mahadev et al. and Adapa et al. demonstrate their fetoscopic techniques, which have similarities but also differences. Mahadev et al. show the remarkable outcome of a fetal repair, with the patient ambulating normally as a young child. Adapa et al. provide additional details regarding the closure of larger defects. Holste et al. describe the use of a mini-laparotomy that further reduces the extent of the surgical procedure experienced by the mother. Finally, Hongsermeier-Graves et al. describe a hybrid approach to fetoscopic closure.

Myeloschisis is the condition when a CSF-containing sac does not elevate the placode from the plane of the spine but is instead flush with the skin. The placode is easier to identify, but the defect in the dura and skin can be more difficult to close. Weber et al. describe their fetoscopic technique to manage this particular situation. Lavadi et al. describe the use of an aspiration tool, the NICO Myriad, designed for confined spaces, as an adjunct to remove an intrauterine hematoma.

The rationale for fetal repair is that early closure of the lumbosacral defect reduces the incidence of secondary features such as hydrocephalus and the development of a Chiari type II malformation. These benefits were described in the MOMS trial but also appear to result from fetoscopic procedures. The primary failure of neural tube patterning and closure, however, likely occurs in the 1st month after conception, and none of the early repairs directly address the impact on spinal cord development. Reynolds et al. describe a procedure performed as part of an ongoing clinical trial evaluating the role of stem cells as a regenerative therapy for the primary failure of neural tube development. This represents the next phase of scientific advancement; rather than just preventing morbidity, can we improve function in this patient population?

Cumulatively, the surgical videos shown in this edition illustrate the rapid change in surgical techniques that are pushing the field of fetal neurosurgery forward. They also identify skills that neurosurgeons who will perform these procedures in the future will need to master.

## Disclosures

R. Bowman reported grants from the Kiwanis Neuroscience Research Foundation that support her research, including the production of this video, during the conduct of the study. M. Zwienenberg reported grants from the California Institute of Regenerative Medicine (CLIN2-15115, co-investigator) outside the submitted work.

## References

[1] Adzick NS Thom EA Spong CY A randomized trial of prenatal versus postnatal repair of myelomeningocele N Engl J Med 2011 364 11 993 1004 10.1056/NEJMoa1014379 21306277 PMC3770179

[2] Sanz Cortes M Chmait RH Lapa DA Experience of 300 cases of prenatal fetoscopic open spina bifida repair: report of the International Fetoscopic Neural Tube Defect Repair Consortium Am J Obstet Gynecol 2021 225 6 678.e1 678.e11 10.1016/j.ajog.2021.05.044 34089698

